# Quality of Life Philosophy VI. The Concepts

**DOI:** 10.1100/tsw.2003.106

**Published:** 2003-12-01

**Authors:** Soren Ventegodt, Niels Jørgen Andersen, Joav Merrick

**Affiliations:** The quality of Life Research Center, Copenhagen, Denmark

**Keywords:** quality of life, QOL, philosophy, human development, holistic medicine, public health, Denmark, Sweden, Norway, Finland

## Abstract

The about a hundred central concepts related to research in the global quality of life can, in a holistic medical frame of interpretation, be organized under ten abstract key concepts: existence, creation of the world, state of being, daily living, talents, relations, sex, health, personal development, and therapy with subthemes as discussed in this paper. The paper shows that the concepts in each group can be seen as related to each other in a quite intuitive and logical way, to give a coherent quality of life philosophy that allows the physician to encourage, inspire, and support his patient. In every consultation, one new concept and idea of existence can be taught to the patient, helping him or her to realize the meaning of life, the source of joy, and the reason for the actual suffering. In this way, we help the patient to mobilize hidden and known resources and to improve quality of life, subjective health, and the ability to function. The concepts were harvested in 2003 at a Nordic seminar on quality of life research, held in Sweden. Life does not only cohere on the inside, but also on the outside. The same power that ties together all the cells in our body, seems to tie us together in relationships and new wholeness. This power evolves into new kinds of relations that unite on more and more complex levels, with the global ecosystem as the highest known level.Our intentions come from this coherent matrix of life. In the beginning of our life, the web of life itself gave birth to our fundamental purpose of life. The abstract purpose determines the frame of interpretation of reality: How we will perceive ourselves throughout life, our inner life, and the world around us. The frame of interpretation is pitched in language and concepts, in fact it creates our perceptions. Based on these perceptions and our purposes of life, our behavior arises. Our consciousness evolves through the witnessing of our behavior and through the response caused by it. Through the slowly acquired mastering of our surrounding world, we obtain our power, which gives us success in life, when we use it responsibly and unite it in harmony with our deepest purpose of life. When many people experience not having success, it is because they are not conscious about their original purpose or the deepest meaning of their lives. They do not know themselves. They do not experience the world in that way and do not realize that they themselves are the cause. Therefore responsibility and self-knowledge, which add up to wisdom, are the ways to a good and successful life.

## INTRODUCTION

Our intention with the development of an explicit quality of life (QOL) philosophy was to obtain the ability to give education and training in philosophy of life to our patients. From pilot studies[1,2] and clinical practice, it seems to have a highly beneficial effect on the patient's quality of life, health, and the ability to function. In order to achieve this goal, it is of the utmost importance to formulate a philosophy of life that is as practical, objective, and supportive of life as possible, and at the same time as free of cultural, religious, and political elements as possible. The more simple, constructive, positive, and intuitive the theory is, the easier it will be understood and the easier it will be to relate to and take possession of by the patient. The clearer and simple the philosophy, the less manipulating.

There are about a hundred fundamental philosophical concepts in the quality of life research; half of them are technical terms related to the tools and technical side of the science, and the other half are related to the meaning and essence of the concepts related to quality of life, being, and existence. The purpose of this article is to give an overview of the last half of the concepts, which is related to the content and meaning of the many quality of life concepts and therefore of central relevance to the patient.

## A HIERACHY OF CONCEPTS

The difficult task is to reduce an enormous philosophical field to something simple and practical. Our approach is to list as many of the concepts as possible, and group them together in a usable hierarchy of concepts, serving our intent of helping the patients improve their own life. This list of core concepts was derived at a Scandinavian course on Quality of Life Research at the Nordic School of Public Health in Gottenburg in 2003. About 20 participants were asked in plenum to list all the concepts they found relevant to the quality of life concept.

According to our quality of life philosophy described earlier[3,4,5,6,7], we selected the abstract concepts used to organize the list of almost a hundred concepts. We ended up with a surprisingly simple, understandable, and useful list of the key concepts of quality of life to be included in and explained by a quality of life philosophy. The presentation of the most central quality of life concepts and the clarification of how these concepts relate to each other is the intent of the present article.

Let us openly admit that our approach is highly subjective, in spite of the many wise people who have contributed to the basic list of concepts, and in spite of all our efforts to set up serious and sober criteria. This article presents the authors' own subjective and philosophical approach, which does not give a conclusive answer to what quality of life is, or how quality of life should be described or perceived. On the other hand, we consider this exercise important because we need to have a clear message for the patients if we wish to teach them and support their philosophical development in another way than just listening to them (which actually is a very efficient approach also).

When we clarify the cornerstones of our philosophy to the scientific society in this manner, we also make it possible for everybody to relate to our work with our patients. Also the patients themselves will get the best possible foundation in order to relate to the philosophy of life we present. The exposition of the philosophy we bring to the patients is therefore also an ethical action. It is important to underline that the article is not meant as a conclusive answer, but more as an encouragement to a dialog, discussion, and ongoing development in the field of philosophy of life.

## THE FUNDAMENTAL CONCEPTS RELATED TO THE QUALITY OF LIFE CONCEPT

During a course in quality of life research at the Nordic School of Public Health, all the participants were given the task to list all the important concepts (in English) related to the concept of quality of life and the concentrated list (that also contains our key concepts) can be seen in Table 1.

**TABLE 1**
**The Fundamental Concepts Related to Quality of Life and the Abstract Key Concepts**

| | | | |
|---|---|---|---|
| Love | Self-expression | Passion | Self-actualization |
| Joy | Understanding | Dependency | The shadow (anti-self) |
| Being | Feeling | Creativity | Near-death |
| Doing | Knowing | Motivation | Resources |
| Life-death | Healing (feel, understand, let go) | Dreams | Honesty |
| Power | Coping | Freedom | Policy |
| Action | Personal development (cope, develop, heal) | Silence | Sex |
| Responsibility | Body | Contemplation | Perspective |
| Success | Good and evil | Belonging | Philosophy of life |
| Purpose | Wholeness | Compassion | Out-of-the-body |
| Intuition | Unity | Caring | Health, ability to function |
| Coherence, intention, purpose and meaning, interpretation and wording, perception, action, consciousness | Spirit/nonlocality | Respect | Psychosomatics |
| Well being | Fragmentation | Awareness | Life pain |
| Satisfaction | Subconsciousness | Acceptance | Competence |
| Happiness | Repression | Acknowledgment | Fight-flee |
| Needs and fulfillment | Empathy | Holding | Life event |
| Talent (gift) | Faith | Dialog | Therapy |
| Existence | Truth | Wonder | Regression |
| Existential therapy | Life, purpose | Awe | Confrontation |
| | Fun | Enlightenment | Innocence |
| | Hope | Wisdom | Choice |
| | Relation | Laughter and sorrow | |
| | Nature | Kindness | |
| | | Pleasure | |
| | | Self/soul and ego | |

The huge amount of concepts cannot be passed on to the patient in a simple way. The list of active concepts in use has to be reduced to a limited number of concepts, organized by a number of abstract key concepts. This is necessary for the concepts to be transferred to the patient's active vocabulary within the limited time of a few consultations. Only with a limited number of very clear and meaningful concepts can we have a simple and useful philosophy that patients can acquire quickly and efficiently, in order to increase their responsibility towards their own life and existence and improve their quality of life, health, and the ability to function. In clinical practice, we use every consultation to introduce a new, central existential concept to the patient. And every time we see the patient, s/he will be given home exercises that facilitate the adaptation of the new concept.

When working with the patient in the clinical practice, some main topics are more relevant than others, hence the choice of key concepts. Table 2 consists of concepts listed under the main topics that we understand as the most important for the holistic medical practice: existence, creation of the world, state of being, relations, daily living, talents, sex, health, personal development, and therapy. When the patient has fully understood these topics and learned to master the concepts as an integrated part of his or her active vocabulary, the patient has often come a long way, and quality of life as measured by a global quality of life questionnaire like QOL5[8] or SEQOL[9] seems often to be radically improved[1,2].

**TABLE 2**
**Quality of Life Related Concepts**

| | | | |
|---|---|---|---|
| **Existence** | Perception | Wisdom | **Personal** |
| Life | Action | Creativity | **Development** |
| Death | Consciousness | **Relations** | Cope, develop, heal |
| Mind | Power | Compassion | The shadow (anti-self) |
| Feelings | Responsibility | Empathy | Resources |
| Body | Success | Faith | Contemplation |
| Love | **States of Being** | Dialog | Perspective |
| Joy | Well being | Policy | Philosophy of life |
| Motivation | Satisfaction | Dependency | Self-expression |
| Being | Happiness | Freedom | Enlightenment |
| Doing | Needs and fulfilment | Kindness | **Therapy** |
| Self/soul and ego | Self-actualization | Honesty | Life event |
| Good and evil | **Daily Living** | Belonging | Confrontation |
| Wholeness | Fun | Sex | Life pain |
| Unity | Hope | Passion | Fight-flee |
| Spirit/nonlocality | Dreams | Pleasure | Near-death |
| Truth | Laughter and sorrow | Innocence | Out-of-the-body |
| Nature | Silence | **Health** | Existential therapy |
| Sex | Wonder | Ability to function | Holding |
| Growth | Awe | Psychosomatics | Care |
| Choice | **Talents** | Healing (feel, | Respect |
| **Creation of the World** | Talent (gift) | understand, let go) | Awareness |
| Coherence | Intuition | Coping | Acceptance |
| Intention | Understanding | Fragmentation | Acknowledgment |
| Purpose and meaning | Feeling | Subconsciousness | Regression |
| Interpretation and | Knowing | Repression | Healing |
| wording | Competence | | |

These topics will therefore be analyzed below, illustrated through the matching concepts. It is important to understand that in this article the concepts points at each other. All in all, the quality of life concepts and their mutual relations set a certain, positive perspective on life and reality. Needless to say, the quality of life concept can be interpreted in many different ways, depending on the agenda, fundamental purpose of the patient, physician, or researcher. A philosophical or a sociological approach tends to emphasize other concepts and themes as central and relevant, than our medical approach. Since quality of life can also be understood as a speechless flow of life, our verbal approach is of course limited by it being a mental and intellectual approach. A playfully intuitive and speechless approach is probable better for the patient in the end, but seldom possible in the beginning. An approach through the mind is therefore often the key for the patient in order to improve their quality of life and subjective health. Let us now go through the abstract key concepts one by one to see the different perspectives they offer to the patient.

## Existence

The first and most fundamental fact concerning the human being is that man is alive, we exist. We are conceived, born, and we will eventually die. We are biological beings, with bodies, feelings, and minds. We consist of a billion cells, which are all living animals and surprisingly we do still appear like a unity. We are conscious and with our consciousness we are able to form our own lives in a surprisingly free way. Our causal consciousness means that first of all we are a spiritual unity.

Our primary motivation comes from love, joy of life, and life energy. We have an inborn intuitive competence. We have the possibility to be ourselves, be something else, an ego[10] far from our real, true, and natural condition. The more we are ourselves, the more loving, joyful, energetic, and competent we become. Our nature is to be and do things. It is joyful and easy to be, if you accept and acknowledge that it is difficult and painful to do.

The art of life is to balance the joy with the pain. When we cannot bear the pain in life, we can instead repress our good, constructive intentions and substitute them with evil and destructive intentions[11]. We are bound to do this as small children[12]. This is why we as adults consist of equally good and bad. Only an ethical consciousness can set us free and make us good[13]. The existential choice becomes our key to happiness.

## Creation of the World

The second fundamental fact is that we as human beings create our own world. How we do this is of the greatest importance for our patients, who often find themselves in a world they completely or partly dislike. In our understanding, the most basic fact of our personal world is that everything is connected. Antonovsky introduced the difficult concept “sense of coherence” for the subjective interpretation of this[14]. The objective, biological reality demonstrates that cells shape us. They can be seen as small animals, which cohere so well that they melt together to make the unity of the human being: the wholeness or the soul.

Life does not only cohere on the inside, but also on the outside. The same power that ties together all the cells in our body, seems to tie us together in relationships and new wholeness. This power evolves into new kinds of relations that unite on more and more complex levels, with the global ecosystem as the highest known level.

Our intentions come from this coherent matrix of life. In the beginning of our life, the web of life itself gave birth to our fundamental purpose of life. The abstract purpose determines the frame of interpretation of reality: How we throughout life will perceive ourselves, our inner life, and the world around us. The frame of interpretation is pitched in language and concepts, in fact it creates our perceptions. Based on these perceptions and our purposes of life, arises our behavior. Our consciousness evolves through the witnessing of our behavior and though the response caused by it.

Through the slowly acquired mastering of our surrounding world, we obtain our power, which gives us success in life when we use it responsibly and unite it in harmony with our deepest purpose of life. When many people experience not having success, it is because they are not conscious about their original purpose or the deepest meaning of their lives. They do not know themselves. They do not experience the world in that way and do not realize that they themselves are the cause. Therefore responsibility and self-knowledge — which add up to wisdom — are the ways to a good and successful life.

## States of Being

Well being, satisfaction, happiness, fulfillment of needs, and self-actualization are terms describing our present state of being. As life has a surface and an existential depth, it is possible to describe all the different stages of being in dimensions according to existential depth and the degree of subjectivity and objectivity, as described in the integrative quality of life theory[15].

## Daily Living

Concepts like fun, hope, dreams, laughter, sorrow, silence, wonder, and awe are all of extreme importance for a good life. Yet it has been very difficult to map these concepts into a sound philosophy of life as they take their offspring from the nonverbal side of man. Let us stress that fantasy, adventure, excitement, curiosity, and playfulness are factors in living a good life of extreme importance.

## Talents

Talent can be seen as a gift from existence or the fundamental fact that everybody is talented. Talent is developed from the specific purpose of life of a person[16] and everybody seems to have a huge talent with the possibility to discover relations to the purpose of life. According to our understanding of life, everybody has every possible talent, but you can only access your talents through your purpose of life. You become talented if you are able to know yourself and are intent on what you want to do, and wholeheartedly.

Unfortunately we are not free, because we have already made our fundamental decision in regard to what our intentions are in this life[11]. So our purpose of life is our primary talent and the key to all our talents. Some talents are bound to our body, feelings, and mind: understanding, feeling, knowing, intuition, wisdom, creativity, and sexuality. These general talents must be transformed into specific work-related competence, developed through action and exercise. The more a person can rely on his specific talent deriving directly from his purpose of life, the more competent and happy will he eventually be.

## Relations

The fourth fundamental fact is that our life is no better than our relations. The relations are more important for the quality of life the closer a person gets to you[17,18]. Trust is very important here, as trust makes it possible for us to open up and receive from others.

Empathy makes it possible for us to meet and understand the other (thou). Dialog makes it possible to grow together and honesty makes the dialog fertile. When we are children or, later in life when we are out of reach with our own existence, we are highly dependant of our relations. As responsible adults, freedom is the basis for the creation of our lives, as we choose our relations. Social qualities can be developed. More kindness makes us better friends and more compassion makes us better fellow human beings. To belong, to be of value, and to make a difference for other people is the real meaning of life for every human being.

## Sex

Apparently there seems to be three dimensions in living a good life: (1) love from me to you; (2) power and consciousness in body, feelings, and mind; (3) joy and pleasure from the body and our life. All this seems to be of decisive importance for our general health, quality of life, and self-expression[16]. Many people suffer from low self-esteem, and only the human being who accepts him- or herself totally — including the body, all of its organs, and its sexuality — will be able to lead a good life. It looks like the life energy fundamentally is polar and that the sexual polarity in reality is the most fundamental aspect of our biological being, in accordance with our creation through sperm and eggs.

## Health

Many of the concepts that are presented as quality of life concepts are in reality concepts that mark health and the ability to function. There is a huge merging between experienced bad thriving and experienced bad health. It is often very difficult for patients to differ between physical and psychological pain, and many depressive patients with chronic diseases will also get rid of their pain when the depression disappears. Helping the patient to cope is a well-known strategy in medicine, but the mere adaptation will often not improve the quality of life in the long run, as personal development and healing are needed. And to feel the need to develop, you need a certain level of chaos, emotional pain, and discomfort.

Healing — understood in a holistic context — is to feel, to understand, and to let go. The importance for existential healing of letting go (of the negative decisions from painful life events) cannot be stressed enough. Many events and the negative beliefs they caused are buried and repressed in the unconsciousness. In general, the patients developing a new, more responsible consciousness is highly beneficial for the quality of life, health, and the ability to function.

## Personal Development

All around the western world it has become very popular to talk about personal development. Unfortunately the concept now covers everything from aura reading and the swinging of a pendulum to the deep and serious work concerning the introspection of the soul and the existence.

What characterizes the deep and sincere work is that man has to confront himself with his shadow side (the anti-self)[13] and get rid of all negative decisions and all accumulated life pain that s/he has collected through the personal history. People who work successfully with themselves experience the hidden and unexploited resources they have. The freeing of the huge personal resources is therefore a mark for the wanted patient-progress in the holistic therapy. The work with many different life perspectives and the increased latitude is another kind of mark. The goal is a positive philosophy of life and enlightenment or reaching the ultimate, natural, fully conscious, and most joyful state of being.

The physician can help his patient to adapt and to cope. He can also help his patient to develop his personality and gradually come to master the difficult task of using himself optimally in this world. With sufficient trust and holding, the patient can heal. He can spontaneously regress to the painful, early gestalts and integrate his personal past. The sign of the patient healing on the most profound level of the soul is self-knowledge and the gift of knowing and feeling the meaning in life.

## Therapy

The fifth fundamental fact is that life is filled with difficult events and occasions that we have to integrate in order to heal our existence. These events invite us to modify ourselves in order to adapt, to get what we need, and to survive. Negative decisions are in reality decisions that we make, overwhelmed by the pain of life, to disclaim the responsibility and escape from trouble. This means that our confrontation with reality descends and instead we become more introverted, neurotic, old, tired, morbid, and sometimes even physically or mentally ill. Chronic stress is the result of many unfinished events, where we did not succeed to win the fight or did not succeed in escaping from the fight. These conflicts still live their own life inside of us, as we are unconscious about them. Life events can be so intense and almost like near-death experiences, where you experience leaving your own body. These intense life events are surprisingly common as most of us experience them through a lifetime and are often very traumatic if the wound is not healed. Even a modest abandonment or maltreatment experienced in our childhood can scar the soul in serious ways. Most people need to do some healing of their soul.

The solution to the general loss of the ability to live seems to be existential healing. Holistic therapy[19] seems to induce a special condition, that we call "being in holistic process". This condition looks and feels a lot like the original crisis in being just as emotionally intense and filled with "energy" (the same level of arousal), but its movement is exactly the opposite. In the crisis you stretch and modify yourself to be less alive, but to survive, while in the process of existential healing you let go of all the negative decisions that made you adapt and you become your real self again.

This is the mysterious healing that has been the goal of the native shaman (the medicine man). It is little understood scientifically speaking. It looks like it happens spontaneous, when both the physician and the patient share the intention of the patient healing from the bottom of the soul. When the patient trusts the physician and accepts the holding as a combination of care, respect, awareness, acceptance, and acknowledgment, the healing happens and the patient's life is once again blessed.

## DISCUSSION

What we did in this paper, one could argue, was not scientific at all, for the following four good reasons:

- We took a Nordic sample of about 20 scientists doing quality of life research or studying the quality of life concepts. We must presume that nonexperts would come up with a different set of concepts that experts.
- We took a sample from the Nordic countries, known to have a very special culture, highly observant of the quality of life. This obviously gives a bias. The sample of concepts would presumably have been very different in a group of countries from Asia or South America, although quality of life research from Asia seem to use very similar concepts[20,21,22]: satisfaction, well-being, self-esteem, purpose in life, hopelessness, depression, self-acceptance, relationship, hope.
- We took these concepts and made a very unscientific process of abstraction guided by a holistic medical perspective. The abstract categories created by this process are highly unlikely to be identical to a similar process guided by another intent or just done by another group of researchers.
- We are not at all representative of the scientific community. It might be that we put to much importance on sex being three adult men, originally from one of the most liberal of the liberal Scandinavian countries, Denmark.

On the other hand, the concepts chosen look very representative when we compare them to the enormous philosophical literature — the titles of the about 10,000 books related to the quality of life subject and to the titles of the about 10,000 related scientific papers done in the area of quality of life, happiness, well being, and existence. We believe that the Nordic countries are actually good for such a study, as the awareness of quality of life in our culture through 20 years or so makes us competent to choose. That we are from a sexually liberal culture might give the study a bias towards sex; on the other hand, will liberation presumably reveal an interest and need more than it will create it? We believe that all human beings deep down have the same nature and that that the concept of global quality of life is related to nature much more that to nurture and culture. Therefore we believe that we, even in spite of obvious, personal idiosyncrasies, represent the human race well enough to make a fair abstraction from the words to the categories. Finally we have been working for many years teaching patients and others quality of life philosophy, so we know what patients needs and likes; therefore at least some wisdom and experience must be contributed by us making the analysis instead of researchers less experienced in the field.

It is difficult to argue for the validity of a philosophy. It is not really a scientific issue, more as we have pointed out before, the basis for science. We felt obliged to reveal our sources, convictions, and to make our quality of life philosophy as explicit as possible. We have done this to our own satisfaction. As we are not impeccable and the global quality of life research is a new branch of the medical science, we ask the reader to please forgive us for all the errors and mistakes we undoubtedly have made in the process.

## CONCLUSION

Research is still scarce when it comes to the relationship between personal philosophy of life and quality of life, health, and ability to function. From a qualitative perspective it seems fair to suggest that when the patient assumes more responsibility of his or her own life, personal resources can be used better and quality of life can be improved. It also seems fair to suggest that such an improvement will influence many other factors of the patient's life like subjective health, both physical and psychological, and the general ability to function.

A constructive and optimistic quality of life philosophy can mobilize hidden resources of the patient. Helping the patient to develop a better personal philosophy of life — what we call "quality of life as medicine" — seems to be one of the best things that the physician can actually do for his patient. Sometimes, when the patient is hopelessly ill, or not ill at all, but poorly functioning, this is the only way to go about it. The physician can, when focusing on the quality of life key concepts and the simple but encouraging perspectives listed above, play an important role in the patient's life, being the one person that inspires the patient to develop a philosophy of life of love and joy, and of exploration of the thousand unseen possibilities every human life contains.

## ACKNOWLEDGMENTS

This study was supported by grants from The 1991 Pharmacy Foundation, as well as by supplementary grants from Goodwill-fonden, the JL-Foundation, E Danielsen and Wife's Foundation, Emmerick Meyer's Trust, the Frimodt-Heineken Foundation, the Hede Nielsen Family Foundation, Petrus Andersens Fond, Wholesaler C.P. Frederiksens Study Trust, Else & Mogens Wedell-Wedellsborg's Foundation and IMK Almene Fond. We gratefully acknowledge the critical scrutiny and expert linguistic assistance of Ib Ravn, Ph.D. and the assistance of philosopher Maximilian Kromann. We are grateful for the opportunity to collect the quality of life concepts kindly offered by professor Bengt Lindstöm and the Nordic School of Public Health (NHV) in Gottenburg, Sweden. The research was approved by the Copenhagen Scientific Ethical Committee under number (KF)V.100.2123/91.

## BIOSKETCHES

**Søren Ventegodt, MD,** is the Director of the Quality of Life Research Center in Copenhagen, Denmark. He is also responsible for a Research Clinic for Holistic Medicine in Copenhagen and is a popular speaker throughout Scandinavia. He has published numerous scientific or popular articles and a number of books on holistic medicine, quality of life, and quality of working life. His most important scientific contributions are the comprehensive SEQOL questionnaire, the very short QoL5 questionnaire, the integrated QOL theory, the holistic process theory, the life mission theory, and the Danish Quality of Life Research Survey, 1991–94 in cooperation with the University Hospital of Copenhagen and the late pediatric professor Bengt Zachau-Christiansen. E-mail: ventegodt@livskvalitet.org. Website: www.livskvalitet.org/

**Niels Jørgen Andersen, MSc,** Professor, Department of Innovation and Economic Organization, Norwegian School of Management. This department conducts research and provides teaching in central topics related to innovation, business development, management of global companies, business history, and economic organization. Research activities within the Department are related to four core subjects within the discipline: business history, cooperative organizations, business development and entrepreneurship, and finally studies of industries with a special focus on the electricity industry. He is also the dynamic chairman of the nonprofit organization Stiftelsen Holistisk Medisin Scandinavia, which aims to support the scientific development, research, and documentation of complementary and holistic medicine in Scandinavia. E-mail: niels.j.andersen@bi.no. Website: www.bi.no/users/fgl93013/

**Joav Merrick, MD, DMSc,** is Professor of Child Health and Human Development affiliated with the Zusman Child Development Center and Division of Community Health at the Ben Gurion University, Beer-Sheva, Israel and presently the Medical Director of the Division for Mental Retardation, Ministry of Social Affairs, Jerusalem and the Director of the National Institute of Child Health and Human Development. He has numerous publications in the field of child and human development, rehabilitation, intellectual disability, disability, health, welfare, abuse, advocacy and prevention. Dr. Merrick received the Peter Sabroe Child Award for outstanding work on behalf of Danish Children in 1985 and the International LEGO-Prize (“The Children’s Nobel Prize”) for an extraordinary contribution towards improvement in child welfare and well being in 1987. E-mail: jmerrick@internet-zahav.net. Website: www.nichd-israel.com
